# Supplementary material for: Tracking Response and Resistance in Acute Myeloid Leukemia through Single-Cell DNA Sequencing Helps Uncover New Therapeutic Targets
Source: Int J Mol Sci. 2024 Sep 17;25(18):10002. doi: 10.3390/ijms251810002 (PMC11432296; doi:10.3390/ijms251810002)
Supplement: Supplementary file 1 [file ijms-25-10002-s001.zip › ijms-3158420-supplementary.pdf]

Table S1. Description of clonal populations of Pt #1. Legend: T1= myelodysplastic syndrome (MDS) diagnosis; T2= acute myeloid leukemia (AML) diagnosis; T3= on-treatment; T4= relapse. Abbreviations: mut. cells= mutated cells; HET= heterozygous mutation; HOM= homozygous mutation

Clone	Pathogenic variants	Time point	Mut cells (n°)	Other cells (n°)	Total cells (n°)	Proportion (%)
C1	TET2^{N191Kfs*4}	T1	137	216	353	38.1
		T2	3	928	931	0.32
		T3	4	667	671	0.6
		T4	22	5,211	5,233	0.42
C2	TET2^{N191Kfs*4} RUNX1^{R201Q}_HET	T1	41	312	353	11.61
		T2	90	841	931	9.67
		T3	13	658	671	1.94
		T4	307	4,926	5,233	5.87
C3	TET2^{N191Kfs*4} RUNX1^{R201Q} _HOM	T1	6	347	353	1.7
		T2	80	851	931	8.59
		T3	18	653	671	2.68
		T4	70	5,163	5,233	1.34
C4	TET2^{N191Kfs*4}; RUNX1^{R201Q} _HOM; FLT3^{D835H}	T1	0	353	353	0
		T2	157	774	931	16.86
		T3	28	643	671	4.17
		T4	27	5,206	5,233	0.52
C5	TET2^{N191Kfs*4} RUNX1^{R201Q}_HET; FLT3^{D835H}	T1	0	353	353	0
		T2	100	831	931	10.74
		T3	17	654	671	2.53
		T4	17	5,216	5,233	0.32
C6	TET2^{N191Kfs*4} RUNX1^{R201Q} _HOM; NRAS^{G12A}	T1	0	353	353	0
		T2	1	930	931	0.11
		T3	3	668	671	0.45
		T4	85	5,148	5,233	1.62
C7	TET2^{N191Kfs*4} RUNX1^{R201Q}_HOM; KIT^{D816H}	T1	0	353	353	0
		T2	7	924	931	0.75
		T3	1	670	671	0.15
		T4	115	5,118	5,233	2.2
C8	TET2^{N191Kfs*4} RUNX1^{R201Q} _HOM; KIT^{D816V}	T1	0	353	353	0
		T2	0	931	931	0
		T3	2	669	671	0.3
		T4	207	5,026	5,233	3.96
C9	TET2^{N191Kfs*4} RUNX1^{R201Q}_HET; KIT^{D816H}	T1	0	353	353	0
		T2	0	931	931	0
		T3	3	668	671	0.45
		T4	61	5,172	5,233	1.17
C10	TET2^{N191Kfs*4} RUNX1^{R201Q}_HET; NRAS^{G12A}	T1	0	353	353	0
		T2	0	931	931	0

		T3	2	669	671	0.3
		T4	61	5,172	5,233	1.17

Table S2. Description of clonal populations of Pt #2. Legend: T1= acute myeloid leukemia (AML) diagnosis; T2= on-treatment; T3= complete remission. Abbreviations: mut. Cells= mutated cells; HET= heterozygous mutation; HOM= homozygous mutation.

**Clone**	**Pathogenic variants**	**Time point**	**Mut cells (n°)**	**Other cells (n°)**	**Total cells (n°)**	**Proportion (%)**
C1	DMNT3A^{R882C} TET2^{N1584Kfs*6} NPM1^{W288Cfs*12} FLT3-ITD^{3nt}	T1	1,926	8,551	10,477	18.38
		T2	0	1,140	1,140	0
		T3	0	12,209	12,209	0
C2	DMNT3A^{R882C} TET2^{N1584Kfs*6} NPM1^{W288Cfs*12}	T1	1,174	9,303	10,477	11.21
		T2	0	1,140	1,140	0
		T3	0	12,209	12,209	0
C3	DMNT3A^{R882C} TET2^{N1584Kfs*6} NPM1^{W288Cfs*12} FLT3-ITD^{12nt}	T1	480	9,997	10,477	4.58
		T2	0	1,140	1,140	0
		T3	0	12,209	12,209	0
